# Psychological health of women who have conceived using assisted reproductive technology in Taiwan: findings from a longitudinal study

**DOI:** 10.1186/s12905-019-0801-7

**Published:** 2019-07-12

**Authors:** Mei-Zen Huang, Chien-Huei Kao, Kuan-Chia Lin, Jiann-Loung Hwang, Shuby Puthussery, Meei-Ling Gau

**Affiliations:** 1 Department of Nursing, National Tainan Junior College of Nursing, 78, Sec.2 Minzu Rd, Tainan City, Taiwan; 2 Department of Midwifery and Women Health Care, National Taipei University of Nursing and Health Sciences, 365, Ming-Te Road, Peitou, Taipei, Taiwan; 3 Institute of Hospital and Health Care Administration, National Yang-Ming University, 155, Sec. 2, Linong Street, Taipei, Taiwan; 4 Department of Obstetrics and Gynecology, Taipei Medical University, 250, Wuxing Street, Taipei, Taiwan; 5 School of Health Care Practice & Institute for Health Research, University of Bedfordshire, Putteridge Bury, Hitchin Road, Luton, Bedfordshire, LU2 8LE UK; 6 Department of Midwifery and Women Health Care, National Taipei University of Nursing and Health Sciences, 365, Ming-Te Road, Peitou, Taipei, Taiwan

**Keywords:** Assisted reproductive technology, Depression, Anxiety, Psychological health, Longitudinal study

## Abstract

**Background:**

Despite the increasing use of Assisted Reproductive Technology (ART) and the significant physical and emotional commitments that these treatments and procedures involve, only limited evidence exists regarding the psychological health of women who undergo ART. This study investigated the changes over time in the psychological health of women who have conceived using ART during the first, second, and third trimesters of pregnancy and during the postpartum period in Taiwan.

**Methods:**

A quantitative longitudinal study was conducted at a fertility centre in Taiwan. 158 pregnant women who had conceived using ART completed a web-based questionnaire that included the following instruments: State Anxiety Inventory, Edinburgh Postnatal Depression Scale, Modified Maternal Foetal Attachment Scale, Pregnancy Stress Rating Scale, Maternity Social Support Scale, Intimate Bond Measure, and Parenting Stress Index. The data were collected the first (9–12 weeks), second (19–22 weeks), third (28–31 weeks) trimesters of pregnancy and at 7–10 weeks postpartum.

**Results:**

Levels of anxiety and depression, which are both key indicators of psychological health, were highest during the first trimester, with scores of 42.30 ± 11.11 and 8.43 ± 4.44, respectively. After the first trimester, anxiety scores decreased and remained stable through the remainder of pregnancy, with scores of 38.03 ± 10.58 in the second and 38.39 ± 10.36 in the third trimester, but increased at two-months postpartum, attaining a score of 41.18 ± 11.68. Further, depression scores showed a similar pattern, declining to a mean of 7.21 ± 4.23 in the second and 6.99 ± 4.11 in the third trimester and then increasing to 8.39 ± 5.25 at two-months postpartum. Pregnancy stress and social support were found to be the most important predictors of change in psychological health during pregnancy and the postpartum period.

**Conclusion:**

Psychological health was found to be poorest during the first trimester and at two-months postpartum. Moreover, pregnancy stress and social support were identified as key predictors of change in psychological health. The findings indicate a need for increased sensitivity among healthcare professionals to the psychological vulnerability of women who have conceived using ART as well as a need to introduce tailored interventions to provide appropriate psychological support to these women.

## Background

Assisted reproductive technology (ART) is a widely accepted and successful approach to enabling couples with untreatable infertility to parent healthy babies. However, ART-related therapies and procedures require significant physical and emotional commitments from the couple, which may impact their psychological health. Studies have shown that women tend to experience anxiety [1, 2], depression [3–6], low self-esteem [7], and dissatisfaction with marital status [5, 8, 9] during the treatment phase. For example, Hjelmstedt et al. [10] found higher levels of anxiety about pregnancy loss among couples who conceived using in-vitro fertilization (IVF) than among couples who had conceived naturally. However, the IVF women in their study viewed their pregnancy more positively and expressed lower levels of concern about the child’s gender and loss of freedom in their future lives as parents than the controls, who had conceived naturally. On the other hand, other previous studies have reported lower or similar levels of anxiety and depression among women who conceived using ART than among women who conceived naturally [8, 11–14]. A systematic review by Verhaak et al. [14] on the psychological health of women undergoing ART found that becoming successfully pregnant using ART was associated with lower levels of anxiety, depression, and stress in new mothers. A longitudinal study by Klock and Greenfeld [11] that compared self-esteem, marital adjustment, and anxiety symptoms in women who had undergone IVF treatment to women who had conceived naturally found that levels of self-esteem, depression, and anxiety were the same in both groups during the 12th and 28th weeks of pregnancy. Furthermore, levels of self-esteem and anxiety in the IVF women increased and decreased, respectively, as their pregnancies progressed. In addition, a more recent study by Raguz et al. [12] reported no statistically significant differences in the proportion of women experiencing depression and anxiety symptoms between a group that had conceived using fertility treatments and a group that had conceived naturally. Other researchers have reported similar findings with respect to levels of depression among parents of singleton babies [11, 13].

Maternal psychological health during pregnancy may impact negatively on the immune system development and functions and the neurocognitive development of the infant [15]. Stress from unhealthy parental and other children’s behaviours, known as parenting stress [16], negatively influences the quality of interactions between the mother and the baby as well as adaptation to the maternal role [17]. Parenting stress has been linked to anxiety and depression both during pregnancy and postnatally [18]. Although factors such as the maternal age, socioeconomic status, social support, history of miscarriage, maternal-foetal attachment, and obstetric complications contribute to maternal stress throughout pregnancy [19], very little is known about the risk factors for psychological health either during pregnancy or postnatally in women who have conceived using ART.

The number of babies born through ART has been increasing over the years in Taiwan and currently account for about 3.03% of annual births [20]. Most of the women who undergo ART treatment are older, with the largest proportion of women between the ages of 34 to 35 years, accounting for 18% of all ART treatments [21]. Although older women may be at higher risk of poor psychological health during both pregnancy and the postnatal period, the psychological health and parenting stress of these women has yet to be sufficiently explored in the literature. Although changes in psychological health over time during the pregnancy and postnatally have been reported in isolated longitudinal studies [10], more evidence is needed regarding how psychological health changes across different periods of pregnancy or the associated factors.

This study aimed to investigate the changes in psychological health over time and the risk factors associated with psychological health during the first, second, and third trimesters of pregnancy and the postpartum period in women who have conceived using ART in Taiwan.

## Methods

This was a quantitative study conducted on a sample of women in Taipei, Taiwan who had conceived using ART. A longitudinal time-series design with repeated measurements of the indicators of psychological health was used.

### Participants

The participants comprised 158 pregnant women who had conceived using ART at a fertility centre in Taipei, Taiwan and given live births. In addition to ART and delivering a live birth, inclusion criteria were: primiparous pregnancy, no history of pre-pregnancy medical complications (e.g., high blood pressure, diabetes, heart disease), and ability to communicate verbally in Mandarin and read/write in Chinese). Eligible women were recruited and baseline data were collected during the first trimester of pregnancy (9–12 weeks). Subsequent data collections were conducted during the second (19–22 weeks) and third (28–31 weeks) trimesters and at 7–10 weeks postpartum. Participants were excluded from this study in the second and third trimesters if they decided to terminate the pregnancy following screening for Down syndrome or other congenital anomalies or if they experienced a premature birth (earlier than the 28th week of pregnancy). In addition, participants whose pregnancies ended in miscarriage or still birth or whose baby died following birth were also excluded from the final analysis.

The required sample size was calculated using G*Power statistical package version 3.1.1 (http://www.gpower.hhu.de/en.html) [22] and estimated as 107 based on the following assumptions: α = .05, power = .80, and effect size = .15. Taking into account a live birth rate of 73.62% following ART [21] and an attrition rate of 10%, the final sample size for enrolment was determined to be 180 women. A total of 187 women at 9–12 weeks of pregnancy were recruited, with 179, 175, and 168 completing the questionnaire at 9–12 weeks, 19–22 weeks, and 28–31 weeks of their pregnancy, respectively. After a further loss to analysis of 10 women, 158 participants completed the questionnaire at 7–10 weeks postpartum (Table 1).

**Table 1:** Characterises of participants

	Pregnant for 9–12 weeks (*N* = 179) n (%)	Pregnant for 19–22 weeks (*N* = 175) n (%)	Pregnant for 28–31 weeks (*N* = 168) n (%)	7–10 weeks after delivery (*N* = 158) n (%)
Periods of pregnancy and work after delivery				
Housewife	75 (41.9)	75 (42.9)	84 (50.0)	85 (53.8)
Career women	104 (58.1)	100 (57.1)	84 (50.0)	73 (46.2)
Chinese herbals treatment				
None	52 (29.1)	156 (89.1)	153 (91.1)	
Yes	127 (70.9)	19 (10.9)	15 (8.9)	
Number of live foetuses				
One	96 (53.6)	108 (61.7)	108 (64.3)	103 (65.2)
Two	55 (30.7)	67 (38.3)	60 (35.7)	55 (34.8)
Three	21 (11.7)			
Four	6 (3.4)			
Five	1 (0.6)			
Discomfort during pregnancy				
None	35 (19.6)	64 (36.6)	55 (32.7)	
Yes	144 (80.4)	111 (63.4)	113 (67.3)	
Complications				
None	169 (94.4)	159 (90.9)	147 (87.5)	
Yes	10 (5.6)	16 (9.1)	21 (12.5)	
Type of complication				
Vaginal bleeding	10 (5.6)	12 (6.9)	9 (5.4)	
UC		5 (2.9)	13 (7.7)	
Placenta previa		2 (1.1)	2 (1.2)	
UTI		1 (0.6)		
GDM			6 (3.6)	
PIH			1 (0.6)	

*UC* Uterine contraction

*UTI* Urinary tract infection

*GDM* Gestational Dibetes Mellitus

*PIH* pregnancy-induced hypertension

After gaining ethics approval from the ethics committee in Taiwan, the principle investigator (MZH) approached potential participants with an information leaflet. Those who after the initial discussion expressed interest in participating were given detailed information about the study and the requirements of participation. Written consent was taken before each participant was enrolled in the study.

### Instruments

The study instruments included the Modified Maternal Foetal Attachment Scale, Pregnancy Stress Rating Scale, State Anxiety Inventory, Edinburgh Postnatal Depression Scale, Maternity Social Support Scale, Intimate Bond Measure, and Parenting Stress Index. Data were collected using a web-based questionnaire that included all of these instruments. The Modified Maternal Foetal Attachment Scale [23] consists of 39 questions that measure maternal foetal attachment using a 5-point scale, ranging from always (5 points) to never (1 point). The Pregnancy Stress Rating Scale [24, 25] is used to measure psychological stress felt by women using a 5-point scale, ranging from never (1 point) to very extreme (5 points). The State Anxiety Inventory [26] is used to measure degree of anxiety on a 4-point scale, ranging from never (1 point) to very (4). The Edinburgh Postnatal Depression Scale [27] is used to screen for degree of depression in pregnant and postnatal woman. The Maternity Social Support Scale [28] includes questions on maternal social support. The Intimate Bond Measure uses a total of 24 questions on two subscales to measure care and control in intimate relationships between couples, with scores ranging from completely incorrect (0) to very correct (3) [29]. The Parenting Stress Index /Short Form (PSI/SF) [16, 30] is designed to assess the interaction-related problems between children and parents. This instrument addresses the theme areas of parental distress, dysfunction in parent-child interactions, and child behavioural difficulties and includes 21 questions that are scored on a 5-point scale ranging from extremely disagree (1) to extremely agree (5). In addition, a structured questionnaire was used to collect general sociodemographic, obstetric, and infant-related information from participants.

The participants were given the choice to complete either the online or printed version of the questionnaire. Nearly all (93.85%) of the participants chose to complete the questionnaire online. Following their enrolment in the study, the principal investigator (MZH) created a secure, password-protected, Internet-based account for each of the participants with an account number and passcode that corresponded to the expected date of birth. The participants were sent their account number and passcode by e-mail. Personal information such as name and address was stored separately. When the expected date of childbirth for a participant was entered, the system automatically produced four different time points for longitudinal data collection at 9–12 weeks of pregnancy, 19–22 weeks of pregnancy, 28–31 weeks of pregnancy, and 7–10 weeks postpartum. In addition, the online account sent email reminders to participants automatically if the questionnaire had not been returned within one week of the data collection time point. If the questionnaire had not been returned by the 14th day after the data collection time point, the lead researcher personally reminded the participant by phone. In order to minimise missing values, the system was set up to mark incomplete answers to remind participants.

### Statistical analysis

The data were summarized as mean and standard deviation values for continuous variables and as proportions for categorical variables. The chi-square test, Student’s t-test, ANOVA, and Pearson’s correlation coefficient were used to analyse associations with *p* values, with <.05 regarded as significant. The three longitudinal models used in this study were the baseline tracking model, repeated time-adjusting/time-lag model, and change model. The generalised estimating equations (GEE) approach was used as a non-parametric method to conduct analyses of repeated measurements [31].

## Results

### Sociodemographic characteristics of the participants

As shown in Table 1, the participants were between 24 and 43 years of age (mean: 34.53 years) and all were married and in heterosexual relationships with a mean relationship duration of 4.75 years. Although a great majority (83.2%) were employed prior to receiving the fertility treatment, 41.9, 42.9, and 50% self-reported as ‘housewife’ during the first, second, and third trimesters, respectively, and 53.8% self-reported as ‘housewife’ at 7–10 weeks postpartum.

Nearly two-thirds (60.3%) of the participants were pregnant for the first time with the remainder reporting a history of assisted miscarriage (24%) or natural miscarriage (15.1%). In addition, small number (6.1%) reported a history of ectopic pregnancy and one participant (0.6%) reported a history of premature birth. The reported fertility problems included male partner-related factors (30.7%); unexplained (30.2%); obstruction of oviduct (27.4%); problems with ovulation (21.2%); and uterine problems (10.6%). Most of the participants (72.1%) transferred four embryos (72.1%), 61.7% had received screening tests for chromosomal abnormalities, and 17.1% had undergone surgery for foetal reduction. Moreover, a large majority reported discomfort during pregnancy, with 80.4, 63.4, and 67.3% reporting problems such as nausea and vomiting, abdominal fullness, urinary frequency, constipation, and back pain during the first, second, and third trimesters, respectively.

Nearly three-quarters (72.8%) of the participants had a full-term delivery, most (65.2%) delivered a singleton birth, and the average singleton birth weight was 2987.66 g. Two-thirds of births were caesarean section (67.1%) due to reasons including delivering twins (48.1%), malpresentation (30.2%), prolonged labour (14.2%), foetal distress (12.3%), elective (8.5%), placenta previa (4.7%), previous myomectomy (2.5%), and ‘giant baby’ (1.9%). Of the participants (32.9%) who delivered normally, 40.4% had an epidural analgesia.

All of the participants followed the Chinese cultural practice, known as ‘doing the month’, of being confined to the home or indoors with reduced physical activity for 1 month following childbirth. More than half (52.5%) did this at a postpartum care centre. Further, although a great majority (69.0%) of women stated during their pregnancy an intent to breastfeed exclusively, 67.1 and 66.5% reported mixed feeding prior to hospital discharge and at one-month postpartum, respectively.

### Psychological health during pregnancy and the postpartum period

#### Anxiety and depression

As shown in Table 2, anxiety scores were highest in the first trimester, with a mean score of 42.30 ± 11.11. There was a reduction in mean anxiety scores after the first trimester to 38.03 ± 10.58 and 38.39 ± 10.36 in the second and third trimester, respectively, and a subsequent rise to 41.18 ± 11.68 during the postnatal period. Using EPDS ≧10 as the cut-off score for depression, the first trimester and the postpartum period had, respectively, the largest and second-largest percentages of participants with depression (36.9 and 34.8%, respectively). Similarly, mean depression scores were highest during the first trimester (8.43 ± 4.44), decreased respectively to 7.21 ± 4.23 and 6.99 ± 4.11 in the second and third trimesters and then rebounded to 8.39 ± 5.25 during the postpartum period.

**Table 2:** Changes in anxiety, depression, intimate relationship, social support, pregnancy stress, and maternal foetal attachment and parenting stress

	First survey (*N* = 179) *M ± SD* (*n*, %)	Second survey (*N* = 175) *M ± SD* (*n*, %)	Third survey (*N* = 168) *M ± SD* (*n*, %)	Fourth survey (*N* = 158) *M ± SD* (*n*, %)
Anxiety	42.30 ± 11.11	38.03 ± 10.58	38.39 ± 10.36	41.18 ± 11.68
Depression	8.43 ± 4.44	7.21 ± 4.23	6.99 ± 4.11	8.39 ± 5.25
≦9	(113, 63.1)	(130, 74.3)	(122, 72.6)	(103, 65.2)
≧10	(66, 36.9)	(45, 25.7)	(46, 27.4)	(55, 34.8)
Intimate relationship				
care	25.34 ± 7.24	26.23 ± 6.81	25.53 ± 7.36	23.92 ± 8.09
control	8.63 ± 5.97	8.67 ± 6.00	8.10 ± 5.25	8.68 ± 6.19
Social support	26.22 ± 3.12	26.34 ± 2.78	26.15 ± 2.83	25.28 ± 3.43
low support	(8, 4.5)	(4, 2.3)	(3, 1.8)	(6, 3.8)
medium support	(36, 20.1)	(37, 21.1)	(48, 28.6)	(55, 34.8)
adequate support	(135, 75.4)	(134, 76.6)	(117, 69.6)	(97, 61.4)
Pregnancy stress	82.92 ± 21.92	83.92 ± 22.07	87.48 ± 22.83	
Foetal attachment	117.03 ± 27.38	134.27 ± 23.34	144.81 ± 21.71	
Parenting stress				48.38 ± 14.50

#### Intimate relationship and maternal social support

The care dimension scores for intimate relationship remained stable across the first, second, and third trimesters with mean scores of 25.34 ± 7.24, 26.23 ± 6.81, and 25.53 ± 7.36, respectively. However, there was slight decrease with respect to care dissension after delivery with a score of 23.92 ± 8.09 points. The control dimension scores for the first, second, and third trimester and the postpartum period were 8.63 ± 5.97, 8.67 ± 6.00, 8.10 ± 5.25, and 8.68 ± 6.19, respectively. Social support appeared stable during pregnancy and during the postpartum period, with mean scores of 26.22 ± 3.12, 26.34 ± 2.78, 26.15 ± 2.83, and 25.28 ± 3.43 in the first, second, and third trimesters and the postpartum period, respectively (Table 2).

#### Pregnancy stress, maternal foetal attachment, and parenting stress

Pregnancy stress increased as the pregnancy progressed, with mean scores of 82.92 ± 21.92, 83.92 ± 22.07, and 87.48 ± 22.83 in the first, second, and third trimesters, respectively. Moreover, maternal foetal attachment showed a steady increase over time, with mean scores of 117.03 ± 27.38, 134.27 ± 23.34, and 144.81 ± 21.71 during the first, second, and third trimesters. Further, parenting stress during the postpartum period earned a mean score of 48.38 ± 14.50 (Table 2).

### Correlates of psychological health

The anxiety score during the first trimester was found to be significantly and positively associated with maternal age (r = .17, *p* < .05), history of artificial abortion (t_(177)_ = − 2.43; *p* < .05), and pregnancy stress (r = .27, *p* < .001) and to be significantly and negatively associated with social support (r = −.32, *p* < .001) and the intimate relationship (r = −.22, *p* < .01) and maternal foetal attachment (r = −.21, *p* < .01) facets of the care dimension. The anxiety score during the second trimester was found to be significantly and positively associated with the length of time spent trying to conceive (r = .15, *p* < .05) and pregnancy stress (r = .35, *p* < .001) and to be significantly and negatively associated with social support (r = −.31, *p* < .001) and the intimate relationship (r = −.22, *p* < .01) and maternal foetal attachment (r = −.18, *p* < .05) facets of the care dimension. In the third trimester, anxiety was found to be significantly and positively associated with absence of a history of ectopic pregnancy (t_(166)_ = 3.31; *p* < .01), planned Caesarean delivery (t _(166)_ = − 2.13; *p* < .05), pregnancy stress (r = .44, *p* < .001), and the intimate relationship facet of the control dimension (r = .23, *p* < .01) and to be significantly and negatively associated with social support (r = −.46, *p* < .001), maternal foetal attachment (r = −.26, p < .01), and the intimate relationship facet of the care dimension (r = −.28, *p* < .001). In the postpartum period, anxiety was significantly higher among participants with a history of artificial abortion than among those without this history (t_(156)_ = − 2.31*p* < .05) and significantly higher among participants who were employed than among those who were housewives (t_(156)_ = 2.26; *p* < .05). Other positive correlates of anxiety found in this study include duration of marriage (r = .18, *p* < .05), the intimate relationship facet of the control dimension (r = .19, *p* < .05), and parenting stress (r = .71, *p* < .001). Negative correlates to anxiety found in the postnatal period include social support (r = − 0.44, *p* < .001) and the intimate relationship facet of the care dimension (r = −.27, *p* < .001).

Positive correlates of depression scores identified in the first trimester include pregnancy stress (r = .26, *p* < .01) and the intimate relationship facet of the control dimension (r = .20, *p* < .01), while the negative correlates include social support (r = −.43, *p* < .001) and the intimate relationship facet of the care dimension (r = −.22, *p* < .01). In the second trimester, housewives had significantly higher scores for depression than their employed peers (t_(173)_ = 2.14; *p* < .05). Moreover, depression scores were significantly and positively associated with length of time spent trying to conceive (r = .19, *p* < .05) and pregnancy stress (r = .37, *p* < .001) and significantly and negatively associated with social support (r = −.42, *p* < .001) and the intimate relationship facet of the care dimension (r = −.28, p < .001). Depression scores in the third trimester were significantly and positively associated with number of foetuses transferred (r = .19, *p* < .05), pregnancy stress (r = .45, *p* < .001), and the intimate relationship facet of the control dimension (r = .35, *p* < .001) and significantly and negatively associated with social support (r = −.50, *p* < .001) and the intimate relationship facet of the care dimension (r = −.30, *p* < .001).

### Temporal changes in anxiety and depression in the tracking models

The anxiety level at two-months postpartum was significantly higher than the anxiety level in the second trimester, both in the baseline tracking model [β: 3.09, 95% CI = 1.33~4.85] and time lag model [β: 5.68, 95% CI = 3.04~8.33]. The factors found to relate significantly to changes in anxiety between the second trimester and the postpartum period were pregnancy stress and maternal foetal attachment, with anxiety levels increasing with increased pregnancy stress [β: 0.27, 95% CI = 0.14~0.39] and decreased maternal foetal attachment [β: -0.06, 95% CI = − 0.12~ − 0.01]. In the time lag model, anxiety levels increased with increased pregnancy stress [β: 0.17, 95% CI = 0.08~0.26] and decreased with increased use of Chinese herbals [β: 3.69, 95% CI = 1.62~5.76].

In the change model, the difference in anxiety level between the first and second trimester was less than that between the third trimester and two-months postpartum. Moreover, the anxiety level increased between the third trimester and the postnatal period [β: 5.95, 95% CI = 3.81~8.09]. The difference in anxiety levels between the first and second trimesters was less than the difference between the second and third trimesters [β: 3.40, 95% CI = 1.37~5.43]. The factors found to correlate significantly with changes in anxiety level were: number of foetuses transferred [β: 5.36, 95% CI = 0.03~10.69], age of the mother [β: -0.18, 95% CI = -0.34~ − 0.03], pregnancy stress [β: 0.17, 95% CI = 0.09~0.25], and social support [β: -0.79, 95% CI = -1.16~ − 0.42] (Table 3).

**Table 3:** GEE estimates^1^ of three longitudinal models for factors associated with state anxiety during four repeat follow-ups

	Longitudinal model 1 (Baseline tracking model)		Longitudinal model 2 (Time-lag model)		Longitudinal model 3 (Change model model)	
Parameters	β	95% CI	β	95% CI	β	95% CI
Non-first time pregnancy vs. First time pregnancy	−1.63	−4.11~0.85	−0.58	−3.04~1.88	− 0.89	− 2.08~0.29
Occupation (Housewife vs. Career women)	− 0.53	− 3.06~2.00	− 1.76	−3.95~0.42	–	–
Chinese herbals treatment (Yes vs. No)	− 0.07	− 2.80~2.66	3.69	1.62~5.76***	–	–
Discomfort during pregnancy (Yes vs. No)	2.27	− 0.43~4.97	1.49	− 0.39~3.38	–	–
Time effects (reference: second trimester)						
Third trimester	0.36	− 0.75~1.47	3.20	1.14~5.25**	3.40	1.37~5.43***
2 months postpartum	3.09	1.33~4.85***	5.68	3.04~8.33***	5.95	3.81~8.09***
Number of fetuses ^3^						
(reference: single)						
Twin	−0.58	−3.39~2.22	0.56	−1.79~2.92		
≧ Triplets	0.24	−3.17~3.65	1.05	−2.22~4.32		
Fetal reduction (reference: fetal reduction ≧2)						
No fetal reduction					7.11	2.22~12.00**
One fetal reduction					5.36	0.03~10.69*
Mother’s age (years)	−0.01	−0.38~0.36	0.15	− 0.18~0.49	− 0.18	− 0.34~ − 0.03*
Duration of the marital relationship (years)	0.12	−0.52~0.77	0.29	−0.34~0.91	0.17	−0.05~0.38
Period of attempted conception (years)	0.26	−0.62~1.14	−0.04	− 0.89~0.81	−0.12	− 0.39~0.15
Frequency of IVF	0.58	−0.71~1.87	0.41	−0.76~1.59	0.38	−0.17~0.92
Number of embryos transferred	−0.14	−1.79~1.51	0.08	−1.46~1.63	0.45	−0.41~1.32
Maternity social support	−0.49	−1.17~0.20	−0.37	− 0.81~ − 0.06	−0.79	− 1.16~ − 0.42***
Pregnancy and parenting stress^3^	0.27	0.14~0.39***	0.17	0.08~0.26***	0.17	0.09~0.25***
Intimate relationship						
care dimension	−0.14	−0.38~0.10	−0.04	− 0.22~0.15	−0.02	− 0.21~0.16
control dimension	−0.11	− 0.34~0.13	0.08	− 0.08~0.23	0.06	− 0.14~0.25
Maternal fetal attachment	−0.06	− 0.12~ − 0.01*	−0.02	− 0.06~0.02	–	–

*p < .05, **p < .01, ***p < .001; ^a^ The number of fetus is changed over time due to fetal reduction

^1^ Controlling for time effects

^2^95% Confidence Interval

^3^Pregnancy and parenting stress: including pregnancy and parenting stress. In order to understand the long-term cause-and-effect relationship between stress and anxiety, I further calculate the scores of stress. After making the women’s real pregnancy and parenting stress divided by the total score of the scale, I gain the percentage and then know the degree of stress in three trimesters and after delivery

Depression levels followed a pattern similar to that of anxiety levels in the long-term tracking models. The depression level at two-months postpartum was significantly higher than that in the second trimester in both the baseline tracking model [β: 1.21, 95% CI = 0.51~1.91] and the time lag model [β: 1.75, 95% CI = 0.78~2.72]. The factors that were found to relate to changes in depression between the second trimester and the postpartum period were: pregnancy discomfort, pregnancy stress, and social support, with depression levels increasing with increased pregnancy discomfort [β: 1.21, 95% CI = 0.08~2.33], pregnancy stress [β: 0.08, 95% CI = 0.03~0.13], and decreased social support [β: -0.50, 95% CI = -0.78~ − 0.22]. In the time lag model, depression levels increased with increased pregnancy stress [β: 0.06, 95% CI = 0.02~0.10] and decreased social support [β: -0.30, 95% CI = -0.51~ − 0.09] and decreased with increased use of Chinese herbals [β: 0.92, 95% CI = 0.11~1.73].

In the change model, the difference in depression level between the first and second trimester was lower than that between the third trimester and two-months postpartum [β: 2.29, 95% CI = 1.38~3.20]. The factors found to correlate significantly with changes in depression level were: duration of marriage [β: 0.11, 95% CI = 0.01~0.20], pregnancy stress [β: 0.07, 95% CI = 0.05~0.10], and social support [β: -0.44, 95% CI = -0.63~ − 0.24] (Table 4).

**Table 4:** GEE estimates of three longitudinal models for factors associated with depression during four repeat follow-ups

	Longitudinal model 1 (Baseline tracking model)		Longitudinal model 2 (Time-lag model)		Longitudinal model 3 (Change model model)	
Parameters	β	95% CI	β	95% CI	β	95% CI
Non-first time pregnancy vs. First time pregnancy	−0.55	− 1.52~0.42	− 0.09	− 1.04~0.85	−0.04	− 0.57~0.49
Occupation (Housewife vs. Career women)	−0.07	−1.08~0. 94	− 0.71	−1.61~0.19	–	–
Chinese herbals treatment (Yes vs. No)	0.07	−0.96~1.10	0.92	0.11~1.73*	–	–
Discomfort during pregnancy (Yes vs. No)	1.21	0.08~2.33*	0.58	−0.17~1.34	–	–
Time effects (reference: second trimester)						
Third trimester	−0.15	− 0.63~0.32	0.57	− 0.22~1.37	0.75	− 0.14~1.64
2 months postpartum	1.21	0.51~1.91***	1.75	0.78~2.72***	2.29	1.38~3.20***
Number of fetuses ^3^						
(reference: single)						
Twin	−0.28	−1.32~0.76	0.42	−0.55~1.39		
≧ Triplets	0.42	−0.96~1.80	0.85	−0.72~2.41		
Fetal reduction (reference: fetal reduction ≧2)						
No fetal reduction					0.27	−2.28~2.81
One fetal reduction					0.06	−2.91~2.79
Mother’s age (years)	−0.12	−0.28~0.04	−0.07	− 0.21~0.08	−0.03	− 0.10~0.04
Duration of the marital relationship (years)	0.15	−0.10~0.40	0.18	−0.05~0.41	0.11	0.01~0.20*
Period of attempted conception (years)	0.05	−0.32~0.42	−0.01	− 0.35~0.34	−0.01	− 0.15~0.13
Frequency of IVF	0.46	−0.11~1.02	0.45	−0.10~1.01	0.16	−0.15~0.48
Number of embryos transferred	0.42	−0.26~1.10	0.54	−0.08~1.17	0.19	−0.23~0.60
Maternity social support	−0.50	−0.78~ − 0.22***	−0.30	− 0.51~ − 0.09**	−0.44	− 0.63~ − 0.24***
Pregnancy and parenting stress^3^	0.08	0.03~0.13**	0.06	0.02~0.10**	0.07	0.05~0.10***
Intimate relationship						
care dimension	0.00	−0.11~0.10	−0.01	− 0.09~0.07	0.00	− 0.09~0.09
control dimension	−0.03	− 0.12~0.06	0.03	− 0.04~0.10	−0.01	− 0.09~0.07
Maternal fetal attachment	−0.01	− 0.03~0.02	0.00	− 0.02~0.02	–	–

*p* *< .05, **p < .01, ***p < .001

## Discussion

This study aimed to examine the psychological health of women in Taiwan who had conceived using ART. As very few studies have focused on longitudinal changes in the psychological health of pregnant women after receiving ART treatments across the three trimesters and during the postnatal period, it is difficult to compare the findings of this study with those of previous studies. Nevertheless, these findings offer a unique opportunity to explore the longitudinal dimensions of psychological health in these participants.

The results show that levels of anxiety were highest in the first trimester and that they remained stable in the second and third trimesters, implying that anxiety lessens as pregnancy progresses. The changes in anxiety reported in our study are consistent with findings from other studies that reported a reduction in anxiety with pregnancy progression [10, 11]. For example, Klock and Greenfeld [11] found lower levels of anxiety in the third trimester than the first trimester among infertile women who had conceived using ART [11]. However, other studies have reported contradictory findings. For example, Da Costa et al. [32] investigated anxiety in women who conceived naturally across the three trimesters and found that anxiety levels rose as pregnancy progressed. The positive correlation between anxiety and maternal age found in this study may reflect the advanced maternal age of most (62%) of the sample, which may be a risk factor for pregnancy failure. Increased maternal age reduces the rates of successful conception and live births following ART treatment and increases the risk of foetal chromosomal abnormalities. Moreover, prior history of pregnancy failure further reduces the chances for conception in older women. All of these factors offer possible explanations for the highest level of anxiety being found in the first trimester of pregnancy. In addition, anxiety was found to increase again during the postpartum period. Hammarberg et al., [33] argued that parents conceiving through ART likely idealise their future roles as parents, which may help explain why anxiety increased after delivery among the participants in this study. As mentioned above and as recommended by Lau et al. [34], this study used a score of ≧10 on the Edinburgh Postnatal Depression Scale as the cut-off point for depression. Although depression scores fell with the progression of pregnancy, they rose again during the postpartum period for reasons that may be the same or similar to that described above with regard to anxiety levels. As no previous studies have used the Edinburg postnatal depression scale to track depression in pregnant women who have conceived using ART treatment across the three trimesters of pregnancy, it is currently not possible to compare the results of this study with the results of other, similar studies. Using the Beck Depression Inventory, Lee et al. [35] found a 25% prevalence of postpartum depression among mothers who had conceived using ART. The even higher prevalence of postnatal depression found in this study may be due to the different measurement methods used.

The stability over time in the intimate relationship facet of the care and control dimensions may be due to the pregnancy easing the tensions associated with infertility and thus strengthening the intimate relationship. However, the decrease in related scores after delivery suggests that child rearing responsibilities and parenting stress weaken the intimate relationship. Klock et al. [11] found that degree of marital satisfaction among women who had conceived using ART was apparently lower than that of women who had conceived naturally. It has also been argued that birth of the first baby may inevitably strain the relationship of the new parents [36].

In this study, pregnancy stress among the participants increased as the pregnancy advanced, with the highest level of pregnancy stress recorded in the third trimester. Previous studies have reported higher levels of pregnancy stress in the first and third trimesters in samples of women who had conceived naturally [32]. Increasing pregnancy stress may be attributed to the physical and psychological changes that women experience as pregnancy progresses and to worries about parenting. ART mothers are more likely to experience pregnancy-related complications such as high blood pressure, abruptio placentae, placenta previa, premature birth, and extremely premature birth than their naturally conceiving peers [37]. The increase in maternal foetal attachment that was found among the mothers in this study is consistent with findings of previous studies [38, 39]. Mothers in this study reported relatively high levels of parenting stress. Tai [30] reported similarly high levels of parenting stress among mothers whose premature baby had left the hospital within one week. The high levels of parenting stress among the mothers in this study may be due to the relatively high ideal-parenting expectations of couples who conceive using ART [33], which may hinder adaption to parenthood and confidence building.

In this study, anxiety levels during all pregnancy trimesters and during the postnatal period were found to significantly and positively correlate with pregnancy stress. Similar findings have been reported by Littleton et al. [40]. In the change model, the difference in anxiety levels between the three trimesters of pregnancy and two-months postpartum is linked to social support. In this study, low social support in the preceding trimester was linked to higher levels of anxiety in the current trimester or postpartum period, implying that social support has a delaying effect on anxiety. However, this effect was apparent only in the change model, and more studies are needed to support the long-term, cause-and-effect relationship between anxiety and social support. Moreover in this study, maternal foetal attachment was identified as a protective factor against pregnancy stress, with higher maternal-foetal attachment in the first trimester associated with lower anxiety in the subsequent trimesters. However, the participants in this study who showed relatively high maternal-foetal attachment in the first trimester were relatively younger and thus had a naturally higher chance of a successful pregnancy. In addition, a significant association between increasing maternal foetal attachment and anxiety was not found in the time lag model. Thus, more studies are needed in order to establish the cause-effect relationship between foetal attachment and anxiety. The participants in this study who received a combined treatment of ART and Chinese herbal reported a higher degree of anxiety in the time lag model during the initial stages of pregnancy, which may be due to the impact of prior miscarriage experiences, pregnancy discomfort, or complications from either of the treatments. However, as the number of participants who received Chinese herbal treatment decreased sharply as the pregnancy progressed, the long-term cause-and-effect relationship during pregnancy between the combined treatment of Chinese herbal and anxiety could not be determined.

The link that was identified in this study between social support and depression during the different stages of pregnancy and the postnatal period is consistent with the findings of other studies [35, 41–49]. The findings of the three long-term tracking models in this study further support the protective effect of social support on postpartum depression. Further, the findings in this study on the associations between pregnancy stress and depression reflect those of previous studies [35, 44, 50, 51] and the findings from the three long-term tracking models indicate that a high level of pregnancy stress in the preceding trimester tends to increase depression in the current trimester. This link between depression and pregnancy discomfort is also supported by prior research. For example, in one study, pregnant women with five or more symptoms of physical discomfort experienced 3.13 times more symptoms of depression than their peers with fewer symptoms [52]. As shown in the baseline tracking model, the participants with pregnancy discomfort in the first trimester reported experiencing higher depression in pregnancy. However, in the time lag model, pregnancy discomfort was a non-risk factor for depression. This indicates that the long-term, cause-and-effect relationship between pregnancy discomfort and depression at different stages of pregnancy cannot be fully understood from this study. In future studies, researchers should take into account changes in pregnancy discomfort and the impact of these changes on depression in pregnancy. Furthermore, postnatal depression was found to increase with duration of marriage. Other researchers have found depression levels among couples with a duration of infertility of 1–3 years to be higher than couples with a duration of infertility duration of one year or less [53]. However, no association between depression level and duration of infertility was identified in this study.

## Conclusion

In this study, the key protective and risk factors found to relate to anxiety both during pregnancy and the postnatal period in women who had conceived using ART were: temporal changes, pregnancy stress, social support, maternal-foetal attachment, combined treatment of Chinese herbals, number of foetuses transferred, and maternal age. Moreover, the protective and risk factors found to relate to depression were: temporal changes, pregnancy stress, social support, pregnancy discomfort, a combined treatment of Chinese herbals, and duration of marriage. Thus, medical and nursing professionals should consider both protective and risk actors when evaluating women’s psychological health. In dealing with women with related risk factors such as a high degree of pregnancy discomfort and pregnancy stress, low social support, and low maternal-foetal attachment, health professionals should screen for symptoms of depression and anxiety throughout the pregnancy and into the postnatal period, and offer appropriate interventions based on the screening results. Future studies should adopt additional long-term tracking models to better assess changes in psychological health and the feasibility of introducing appropriate longitudinal interventions. The instruments used in this study all exhibited good psychometrics properties. The content validity was established using translation and back-translation procedures. The Cronbach’s alpha coefficients were 0.93–0.95 for the State Anxiety Inventory, 0.71–0.76 for the Maternity Social Support Scale, 0.81–0.88 for the Edinburgh Postnatal Depression Scale, 0.93–0.95 for the Intimate Bond Measure, and 0.92–0.94 for the Parenting Stress Index, suggesting that these tools are suitable for use with non-Western subjects.

A major limitation of this study was that participants were recruited from one hospital only. Because different hospitals have different ART consulting resources, the study results are difficult to extrapolate to the general population of ART mothers. As this longitudinal study followed subjects from early pregnancy to the postpartum period, the Modified Maternal Foetal Attachment Scale was applied only during the three trimesters of pregnancy. Thus, the Maternal Foetal Attachment was not included in the GEE change model. Future studies may develop other suitable instruments to measure maternal-foetal/infant attachment for use in longitudinal studies during the perinatal period.

## Data Availability

The datasets used and analysed in this study are available from the corresponding author upon reasonable request.

## Abbreviations

ART: Assisted Reproductive Technology
IVF: in-vitro fertilization
